# Prevalence and Associated Factors of Premenstrual Syndrome Among Female University Students of the Reproductive Age Group in Kabul, Afghanistan: A Cross-Sectional Study

**DOI:** 10.1089/whr.2024.0106

**Published:** 2025-04-10

**Authors:** Zainab Ezadi, Mirwais Ramozi, Hosain Barati, Maryam Hosseini, Shafiqa Hakimi, Nooria Mohammady, Akihiko Ozaki, Yasuhiro Kotera, Yudai Kaneda, Parastoo Ramozi, Basira Bek

**Affiliations:** ^1^ Midwifery Faculty, Khatam Al Nabieen University, Kabul, Afghanistan.; ^2^ Medicine Faculty, Kateb University, Kabul, Afghanistan.; ^3^ School of Medicine, International Campus, Shahid Sadoughi University of Medical Sciences and Health Services, Yazd, Iran.; ^4^ Kabul University of Medical Sciences, Kabul, Afghanistan.; ^5^ Department of Breast and Thyroid Surgery, Jyoban Hospital of Tokiwa Foundation, Iwaki, Japan.; ^6^ Faculty of Medicine and Health Sciences, University of Nottingham, Nottingham, United Kingdom.; ^7^ School of Medicine, Hokkaido University, Sapporo, Japan.; ^8^ Faculty of Psychology and Educational Sciences, Islamic Azad University, Central, Tehran branch, Tehran, Iran.

**Keywords:** premenstrual syndrome, premenstrual dysphoric disorder, Afghanistan, university students, women’s health

## Abstract

**Background:**

Premenstrual syndrome (PMS) is a common menstrual disorder that can significantly impact the physical, emotional, and social well-being of affected women. However, PMS remains a neglected problem in Afghanistan, with limited data on its prevalence and impact. This study aimed to determine the prevalence of PMS and the more severe premenstrual dysphoric disorder (PMDD) among female university students in Kabul, Afghanistan.

**Methods:**

Researchers conducted a cross-sectional study from April to October 2021 at four universities in Kabul. They included a total of 310 female students aged 15–35 years. After data cleaning, 44 participants were removed and 266 participants remained. Data were collected using a validated screening tool for PMS and PMDD, along with information on participants’ sociodemographic and lifestyle factors. Statistical analysis was performed to assess the findings.

**Results:**

The study found that 88.8% of participants screened positive for either moderate to severe PMS (27.1%) or PMDD (61.7%). There was a significant association between lower educational attainment and higher rates of PMS/PMDD.

**Conclusion:**

This study uncovered a high prevalence of PMS and PMDD among female university students in Kabul. These conditions significantly impact young Afghan women’s health and well-being, but remain a neglected issue. Urgent action is needed to improve awareness, screening, and treatment access for premenstrual issues in Afghanistan.

## Introduction

Premenstrual syndrome (PMS) is a cyclical disorder occurring during the late luteal phase of the menstrual cycle. PMS is characterized by physical, emotional, and psychological symptoms starting a week before menstruation and ends by the onset of menstruation.^1,2^ The severe form of PMS is known as premenstrual dysphoric disorder (PMDD).^3,4^ PMS is a common menstrual disorder affecting many reproductive age women, 50%–80% of women of reproductive age have at least mild premenstrual symptoms, ∼30%–40% of women report PMS symptoms that require treatment, and 3%–8% of women have PMDD.^5^ About half of women who need relief from their PMS also have another health problem that may worsen during the premenstrual period.^6,7^ The main cause of PMS is unknown. But various factors are associated with PMS like psychological (stress, post-traumatic stress disorder [PTSD], combat, and natural disasters), sociodemographic (age, marital status, and living region), lifestyle, and dietary factors including habits like fat-rich diet, exercise, alcohol consumption, caffeine beverages, and smoking. Other factors associated with the syndrome include family income, being sexually active, a lengthy menstrual cycle, and age at menarche.^8–10^

The American College of Obstetrics and Gynecology defines the diagnostic criteria for PMS as follows: at least one somatic (headache, abdominal bloating, breast tenderness, swelling of extremities) and affective (depression, anxiety, irritability, angry outburst, and social withdrawal) symptom must be experienced 5 days prior to the onset of menses for three consecutive menstrual cycles and must end within 4 days of the onset of menses.^11,12^

Studies show that women experiencing PMS symptoms are more susceptible to mental health disorders and weakened physical functioning, which can lead to a markedly reduced quality of life, increased rates of workplace and school absenteeism, high rates of suicide and accidents, poor academic performance, deterioration of social and interpersonal relationships, and frequent hospitalization.^13–15^

PMS is a significant issue that affects many young women, according to epidemiological survey 75% of women suffer from PMS symptoms, and 3%–8% suffer from severe symptoms of PMS.^10^ Different countries have shown varying rates of PMS prevalence: 34% in China, 71% in Turkey, 80% in Pakistan, and 92% in Jordan.^16^ But it is overlooked in developing countries like Afghanistan, and there is not any official statistics about the prevalence of PMS. Afghan women face numerous challenges related to menstruation, including limited access to menstrual products, lack of education, poor sanitation, cultural taboos, and gender-based violence. These challenges have a major impact on the physical and mental health of Afghan women, which can be worse during menstruation. Despite the significant impact of PMS on women’s health-related quality of life, it has received inadequate attention. Estimating the overall prevalence of PMS at the country level can provide a more comprehensive picture compared to individual studies. Therefore, this cross-sectional study aimed to determine the prevalence of PMS and its associated factors among female university students in Kabul, Afghanistan.

## Materials and Methods

### Study design and participants

The research has carried out a cross-sectional study among all women aged 15–45 years studying at the universities located in Kabul/Afghanistan. The study spanned 7 months from April to October 2021, and we included 310 women through convenient sampling. An invitation speech highlighted the aim of the study, and the procedures were conducted by researchers in each class for the available participants. Female students who were interested in participating in the study were asked to specific room in their free time to complete the Premenstrual Symptoms Screening Questionnaire (PSST). Participation in the study was voluntary and did not involve financial or other compensation. Women of reproductive age (from 15 to 45 years) with regular menstrual cycles (from 21 to 35 days) were included in the study. Of those who have physical problems such as thyroid disorders, pelvic inflammatory diseases, and those who have used contraceptives were excluded from the study.

### Ethical consideration

Ethical approval for the study was obtained from the Institutional Ethical and Research Committee at Khatam Al Nabieen Private University in Kabul, Afghanistan. Permission to collect data from the female university students was obtained from the principals of the respective colleges. Written informed consent was obtained from all the participants.

### Data collection tool

The PSST, designed to identify women suffering from severe PMS or PMDD, along with a sociodemographic questionnaire, was utilized in this study. The PSST consists of 19 questions divided into two parts. The first part includes 14 questions assessing mood, physical, and behavioral symptoms. The second part measures the impact of these symptoms on a person’s life and consists of five questions (Table 1).

**Table 1.** The Premenstrual Symptoms Screening Tool

Symptoms	Not at all	Mild	Moderate	Severe
Do you experience some or any of the following premenstrual symptoms which start before your period and stop within a few days of bleeding?				
1. Anger/irritability				
2. Anxiety/tension				
3. Tearful				
4. Depressed mood				
5. Decreased interest in work				
6. Decreased interest in home				
7. Decreased interest in social				
8. Difﬁculty concentrating				
9. Fatigue/lack of energy				
10. Overeating				
11. Insomnia				
12. Hypersomnia				
13. Feeling overwhelmed				
14. Physical symptoms: breast tenderness, headache, joint/ muscle pain, bloating, weight gain				
Have your symptoms, as listed above, interfered with:				
A. Work efficiency/productivity				
B. Relationship with co-workers				
C. Relationship with family				
D. Your social life activities				
E. Home responsibility				

For each question, four response options were given: “not at all,” “mild,” “moderate,” and “severe,” corresponding to a score of zero (no symptoms) to three (severe symptoms that prevent daily activities and require pain medication).^17^ According to this questionnaire, the minimum possible score was zero, and the maximum score was 57. We classified a score between 0 and 19 as indicative of mild PMS symptoms, a score between 20 and 28 as moderate PMS symptoms, and a score above 28 as severe PMS symptoms.

To categorize a participant as having mild, moderate, or severe PMS, the following criteria must be met:

1. At least one of the symptoms #1, #2, #3, or #4 must be rated as moderate to severe.
2. In addition, at least four of the symptoms #1-#14 must be rated as moderate to severe.
3. At least one of the symptoms A, B, C, D, or E must be rated as moderate to severe.

On the other hand, PMDD is distinguished from PMS when at least one of the symptoms #1, #2, #3, or #4 and one of the symptoms A, B, C, D, or E are rated as severe.

### Validity and reliability of questionnaire

The validity and reliability of the Farsi version of the questionnaire were established by Siah Bazi et al., with a Cronbach’s alpha of 0.9. The content validity ratio and content validity index were reported as 0.7 and 0.8, respectively.^18^

### Data collection procedures

The study was conducted after obtaining permission from the relevant authorities. The participants were provided a brief explanation of the study, and their informed consent was obtained. Data were collected using a self-administered questionnaire. The questionnaire included information on the participants’ sociodemographic characteristics, menstrual cycle characteristics, and lifestyle factors. Students who were interested to participate in the study were invited to a specific room one by one. No identifier information was collected from participants, and each participant was identified by a numerical code to ensure the privacy and confidentiality of the participants.

### Data analysis

Descriptive and inferential statistics were used for the analysis of data using SPSS version 21.0 as per the study objectives. In descriptive analysis, calculations were done by using frequency and percentage. The chi-squared and one-way analysis of variance tests were used to identify the correlation between PMS, PMDD, and sociodemographic variables. All analysis was performed with a 95% confidence level and a significant level defined as a *p* value ≤0.05.

## Result

Out of 310 participants, 44 participants did not answer the PSST questionnaire completely, hence they were excluded from the analysis, and ultimately 266 participants remained. Sociodemographic characteristics of study participants are summarized in Table 2. The mean age of the participants was 23.34 ± 3.95 years. The majority of participants were single (67.7%), and in the first year of higher education (47.4%). Among participants, 78.9% had regular menstruation while 13.5% reportedly irregular menstruation. Moreover, most of the participants (75.2%) had menstruation ≤6 days in each cycle.

**Table 2.** Sociodemographic Characteristics of Study Participants

Variables	Category	Frequency	Percentage (%)
Age group	18–20	46	17.3
	21–25	122	45.9
	26–35	52	19.5
	Missing	46	17.3
Marital status	Marriage	63	23.7
	Single	180	67.7
	missing	23	8.6
Menstruation status	Regular	210	78.9
	Irregular	36	13.5
	Missing	20	7.5
Menstruation Duration	≤6 days	200	75.2
	≥7 days	50	18.8
	Missing	16	6
Education	first year	126	47.4
	middle years	87	32.7
	last year	16	6
	Missing	37	13.9

In total, 166 (62.4%) participants were screened as having PMDD, while 70 (26.3%) participants were having moderate/severe PMS and 30 (11.27%) were having no or mild PMS. None of the sociodemographic characteristics were associated with PMS, and PMDD condition (*p* > 0.05) except education level (*p* = 0.02) (Table 3). The distribution of specific PMS symptoms across different severity levels is presented in Table 4.

**Table 3.** Sociodemographic-Based Distribution of Premenstrual Syndrome, and Premenstrual Dysphoric Disorder Among Participants

	No/mild PMS		Moderate/sever PMS		PMDD		*P*
	*n*	%	*n*	%	*n*	%	
Age (year)							
18–20	5	16.7	14	20	27	16.3	0.6
21–25	12	40	29	41.4	81	48.8	
26–35	8	26.7	15	21.4	30	18.1	
Missing	5	16.7	12	17.1	28	16.9	
Marital status							
Marriage	5	16.7	18	25.7	40	24.1	0.5
Single	23	76.7	43	61.4	144	68.7	
missing	2	6.7	9	12.9	12	7.2	
Menstruation status							
Regular	25	83.3	51	72.9	134	80.7	0.1
Irregular	4	13.3	14	20	18	10.8	
Missing	1	3.3	5	7.1	14	8.4	
Menstruation duration							
days ≥6	22	73.3	51	72.9	122	73.5	0.9
<7 days	6	20	14	20	30	18.1	
Missing	2	6.7	5	7.1	14	8.4	
Education							
first year	16	53.3	41	58.6	69	41.6	0.02
middle years	9	30	13	18.6	65	39.2	
last year	2	6.7	6	8.6	8	4.8	
Missing	3	10	10	14.3	24	14.5	
Total	30	11.27	70	26.3	166	62.4	

PMDD, premenstrual dysphoric disorder; PMS, premenstrual syndrome.

**Table 4.** Frequency of Premenstrual Syndrome Symptoms by the Level of Severity

Premenstrual symptoms	Not at all*N* (19)	Mild*N* (19)	Moderate*N* (19)	Severe*N* (19)
Depressed mood	36 (13.5)	40 (15)	76 (28.6)	114 (42.9)
Anxiety/tension	37 (13.9)	62 (23.3)	86 (32.3)	81 (30.5)
Tearful	63 (23.7)	49 (18.4)	73 (27.4)	81 (30.5)
Anger/irritability	53 (19.9)	50 (18.8)	75 (28.2)	88 (33.1)
Decreased interest in work	56 (21.1)	50 (18.8)	86 (32.3)	74 (27.8)
Decreased interest in home	48 (18)	73 (27.4)	78 (29.3)	67 (25.2)
Decreased interest in social	65 (24.4)	60 (22.6)	70 (26.3)	71 (26.7)
Difﬁculty concentrating	41 (15.4)	63 (23.7)	80 (30.1)	82 (30.8)
Fatigue/lack of energy	21 (7.9)	55 (20.7)	78 (29.3)	112 (42.1)
Overeating	105 (39.5)	65 (24.4)	52 (19.5)	44 (16.5)
Insomnia	81 (30.5)	50 (18.8)	83 (31.2)	52 (19.5)
Hypersomnia	55 (20.7)	61 (22.9)	77 (28.9)	73 (27.4)
Feeling overwhelmed	65 (24.4)	64 (24.1)	72 (27.1)	65 (24.4)
Physical symptoms	52 (19.5)	52 (19.5)	80 (30.1)	82 (30.8)
Work efficiency/productivity	71 (26.7)	74 (27.8)	77 (28.9)	44 (16.5)
Relationship with co-workers	43 (16.2)	65 (24.4)	101 (38)	57 (21.4)
Relationship with family	41 (15.4)	65 (24.4)	101 (38)	59 (22.2)
Your social life activities	50 (18.8)	80 (30.1)	87 (32.7)	49 (18.4)
Home responsibility	50 (18.8)	48 (18)	98 (36.8)	70 (26.3)

PMS symptoms, with the exception of overeating, were present in over 50% of individuals at moderate to severe levels (Table 5). The most common symptoms were depressed mood (71.5%), fatigue/lack of energy (71.4%), impairment in home responsibilities (63.1%), and anxiety/tension (62.8%) (Table 5). Furthermore, the prevalence of symptoms differed significantly across the three groups: no or mild PMS, moderate/severe PMS, and PMDD (*p* = 0.0001). Additionally, there was a notable increase in symptom prevalence with the severity of PMS (Table 5).

**Table 5.** Frequency Comparison of Symptoms in Three Groups of No PMS/Mild PMS, Moderate to Severe PMS, and PMDD

	No/mild PMS		Moderate/sever PMS		PMDD		*p*
	*N*	%	*N*	%	*N*	%	
Depressed mood	7	23.4	39	55.7	144	86.7	0.0001
Anxiety/tension	5	16.7	29	41.4	133	80	0.0001
Tearful	6	17	26	37.2	122	73.5	0.0001
Anger/irritability	7	23.4	27	38.6	128	77.1	0.0001
Decreased interest in work	11	36.6	34	48.6	122	73.5	0.0001
Decreased interest in home	6	20	23	32.9	116	69.8	0.0001
Decreased interest in social	5	16.6	22	31.4	114	68.6	0.0001
Difﬁculty concentrating	7	23.3	23	32.8	132	79.5	0.0001
Fatigue/lack of energy	12	40	44	62.8	134	80.7	0.0001
Overeating	4	13.3	17	24.2	74	44.7	0.0001
Insomnia	3	10	24	34.3	108	65.1	0.0001
Hypersomnia	7	23.4	32	45.7	111	66.9	0.0001
Feeling overwhelmed	8	26.7	18	25.7	111	66.8	0.0001
Physical symptoms	8	26.7	33	47.1	121	72.9	0.0001
Work efficiency/productivity	4	13.3	20	28.6	97	58.4	0.0001
Relationship with co-workers/ peers	8	26.7	39	55.8	111	66.9	0.0001
Relationship with family	7	23.3	37	52.8	116	69.9	0.0001
Your social life activities	8	26.6	26	37.1	102	61.4	0.0001
Home responsibility	8	26.7	43	61.4	117	70.5	0.0001

## Discussion

This study aimed to assess PMS and PMDD among Afghan female university students to provide evidence-based information to address the condition and its consequences on the overall health and life quality of female university students.

There are no specific data available on the prevalence of PMS among Afghan female students. This study showed that PMDD is common among Afghan students (61.7%) and PMS (27.1%). The prevalence of PMDD and PMS among students globally varies widely. Several studies have reported varying rates of PMS, depending on the definition of PMS used, the study population, and the methodology of the study. Similarly, it appears that the PMDD prevalence differs depending on culture, as well as ethnic group.

A study conducted in the United States reported that up to 85% of women experience at least one symptom of PMS during their menstrual cycle, while up to 5% of women experience severe symptoms that interfere with their daily activities. Another study conducted in China reported that the prevalence of PMS among college students was 25.3%. In Nigeria, a study reported that the prevalence of PMS among university students was 32.5%. In Malaysia, a study reported that the prevalence of PMS among female university students was 91.4%. Zaka et al. showed very high frequency (81.25%) of PMS among women of reproductive age has been reported in Pakistan.^19^

The reasons for the differences in the prevalence of PMS among students in different countries may be related to a variety of factors. These include differences in cultural attitudes toward menstruation, access to healthcare and menstrual products, lifestyle factors such as diet and exercise, and hormonal and even genetic factors. For example, in countries where menstruation is considered a taboo topic including Afghanistan, women are less likely to seek medical attention for symptoms of PMS. In addition, differences in diet and physical activity levels play a role in the prevalence of PMS, as studies show women who eat a healthy, balanced diet and exercise regularly may be less likely to experience PMS symptoms than those who do not.^20,21^ In Afghanistan, strict cultural norms in seeking reproductive healthcare, low level of health education, and large-scale food insecurity are long-lasting challenges that Afghan females face to address their reproductive health.^22^

Finally, genetic factors also play a role in the prevalence of PMS, as studies have suggested that certain genetic variations may increase the risk of developing PMS.^23,24^ However, PMS is a common condition worldwide, and it is likely that some Afghan girls experience it as well. Given the challenging circumstances that many Afghan women face, it is possible that such above-mentioned factors could contribute to the prevalence of PMS among Afghan girls’ students.

The PMDD prevalence in a nationwide sample of Korean women is 2.4%,^25^ 3.3% in the Bulgarian population,^26^ 7.7% among female university students in Jordan,^27^ and even 17.6% in young adult women in southern Brazil. Herein, global and regional studies focusing on the prevalence are required for appropriate and more precise exploration of the global prevalence of PMS/PMDD.^28^

PMS can have a significant adverse impact on a woman’s quality of life and productivity, as well as on her self-confidence, self-esteem, and social life.^29^ The physical symptoms of PMS, such as cramping, bloating, and fatigue, can be debilitating and can interfere with a woman’s ability to carry out daily activities and responsibilities. In addition, the emotional symptoms of PMS, such as irritability, mood swings, and depression, can lead to difficulties in personal and professional relationships and can affect a woman’s ability to concentrate and perform well at work or school.

In this study, the results showed that a high percentage of students suffered from different kinds of menstrual symptoms: depressed mood 114 (42.9%), fatigue/lack of energy 112 (42.1%), and anger/irritability 88 (33.1%). These symptoms have negative impacts on women’s quality of life.

PMS can also have a negative impact on a woman’s self-confidence and self-esteem, particularly if she feels embarrassed or ashamed about her symptoms. Women who experience severe PMS symptoms may feel like they are not in control of their own bodies, which can lead to feelings of helplessness and frustration. In terms of social life, women with PMS may avoid social activities or events during their menstrual cycle because of discomfort or embarrassment particularly in communities with low health education levels such as Afghanistan. As a consequence, consistent with this study, the symptoms interfere with productivity and social life of female students. In addition, PMS can also affect a woman’s sexual health and well-being. Women with PMS may experience a decreased libido, which can affect their relationships and intimacy with their partners. Overall, the adverse impact of PMS on a woman’s quality of life and productivity can be significant. In line with previous studies, this study’s results showed that a high percentage of students suffered from depressed mood 114 (42.9%), fatigue/lack of energy 112 (42.1%), and anger/irritability 88 (33.1%) during their menstrual cycle, with negative impacts on Afghan women’s quality of life. Therefore, it is important for women to seek medical advice and support to be evaluated for this syndrome in order to be timely diagnosed and effectively improved overall well-being. It is important to note that PMS is a treatable condition and there are various strategies that can help to manage symptoms. These may include lifestyle changes such as regular exercise, a balanced diet, and stress reduction techniques, as well as medications such as hormonal contraceptives and pain relievers.

Several sociodemographic variables, such as age, education level, marital status, occupation, socioeconomic status, and ethnicity, have been found to be associated with PMS. This may be due to differences in hormonal levels, as hormonal fluctuations tend to be more erratic in younger women. On the other hand, the association of variables such as education level, marital status, occupation, and socioeconomic status may implicate psychological stress, nutrition, and lifestyle. Stress is a striking factor that can influence the likelihood of experiencing PMS symptoms. Stress can also make existing PMS symptoms worse.^20^ While education level, marital status, or occupations may not be directly related to PMS, it is possible that such variables indirectly associated with PMS symptoms due to work, school and university, or personal issues may be more likely to experience PMS symptoms. A study indicates a high prevalence of depression and anxiety among Afghan women. Similarly, depressive mood was the main symptoms found in this study. Herein, for Afghan women depression and anxiety are the main mental diseases to be addressed. Education may be resultant to higher health knowledge, and access to healthcare resources leads to the lowest level of PMS experience. In the current study, the higher education level is associated with severe PMS. One explanation can be although they have higher knowledge levels, at the same time the higher stress level among Afghan students, and malnutrition complicated by general limitations to access proper public health facilities in Afghanistan result in a higher prevalence of PMS among higher educated women.

Studies have shown that married women are more likely to experience PMS than single or divorced women.^10,30^ This may be due to the stress and demands of marital relationships. Women in certain occupations may be more likely to experience PMS due to job-related stress and demands. The prevalence of PMS may be higher among healthcare workers than among nonhealthcare workers or women in service and management positions had a higher risk of PMS.^10,31–33^

Studies have shown that women with a lower socioeconomic status are more likely to experience PMS than those with a higher socioeconomic status. This may be related to differences in access to healthcare resources and higher levels of stress due to economic challenges. Afghan women living in a country with widespread poverty are the most vulnerable to suffer the consequences.^34^

Overall, these sociodemographic variables can play a significant role in the likelihood of experiencing PMS directly or indirectly. It is important for healthcare providers to be aware of these factors when assessing women for PMS symptoms and providing appropriate support and treatment.

PMS and PMDD are conditions that can have negative effects on students’ academic performance, social relationships, and overall.^33^ PMDD is a severe form of PMS that is characterized by mood disturbances such as depression, anxiety, irritability, and anger. These symptoms can be debilitating and interfere with a student’s ability to concentrate, participate in class, and complete assignments. Women with PMDD were found to have significantly lower academic performance and higher rates of school absenteeism compared to women without PMDD.^35,36^ PMS can also have negative effects on academic performance. Symptoms such as fatigue, headaches, and cramps can make it difficult for students to attend classes, study, and complete assignments. High school girls with PMS were found to have lower academic achievement and more school absences than girls without PMS.^35–37^ In addition to academic performance, PMDD and PMS can also affect students’ social relationships and mental health. Students with PMDD or PMS may experience difficulties in their relationships with friends and family due to mood disturbances and irritability. They may also experience anxiety and depression related to their condition. Women with PMDD were found to have higher rates of depression and anxiety compared to women without PMDD. Consistently, this study found 164 (61.7%) participants with PMDD.

Overall, the consequences of PMDD and PMS among students can be significant and may impact academic performance, social relationships, and mental health. It is important for students who experience these conditions to seek support from healthcare professionals, counseling services, and academic advisors in order to manage their symptoms and maintain their academic and personal goals.

While this research study provides valuable insights into the prevalence of PMS/PMDD among women in Afghanistan, several limitations in sampling and data collection must be considered. These constraints temper the findings and necessitate cautious interpretation. First, the sampling strategy, convenience sampling, although adequate for an initial analysis in a group of university students, is unlikely to be representative enough to capture the diverse sociodemographic factors among Afghan women that influence PMS/PMDD prevalence. Moreover, focusing on a specific population limits the generalizability of the findings to broader populations. Second, the reliance on self-reported information from study participants, without a confirmatory mechanism for PMS symptoms, may result in underestimation or overestimation of the prevalence of PMS. Finally, participants were provided with a brief explanation of the study prior to giving consent, which may have influenced participation rates, particularly among those with and without PMS/PMDD symptoms, potentially increasing the number of participants with symptoms. This could lead to a significant overestimation of PMS/PMDD prevalence. These limitations may constrain a comprehensive understanding of PMS/PMDD prevalence in the study population. Hence, while this study offers valuable insights, these limitations highlight the need for future research to address broader population diversity, risk factor assessment, and management. Additionally, efforts should be directed toward appropriate interventions to raise awareness, improve access to treatment, and provide support for those affected.

## Conclusion

This study among female university students in Kabul, Afghanistan, found that a high proportion—over 88%—of participants screened positive for either moderate to severe PMS or the more severe PMDD. The research also found a significant link between lower educational attainment and higher rates of PMS/PMDD. These findings underscore the substantial burden that premenstrual disorders place on young Afghan women, which can negatively affect their academic performance, mental health, and overall quality of life. However, PMS remains a neglected issue in Afghanistan, with limited awareness, inadequate access to treatment, and lack of supportive policies. A potential first step for intervention could be the implementation of awareness campaigns, similar to those successfully introduced in countries like Malaysia and Pakistan, where education about PMS and PMDD in universities and community settings has proven effective in improving understanding and access to care. Such campaigns in Afghanistan could be targeted at both students and healthcare providers, focusing on early detection, management, and treatment options. This should be accompanied by improved education, screening programs, and provision of appropriate medical and psychological care. Further research is also warranted to better understand the risk factors and lived experiences of Afghan women suffering from premenstrual disorders. Addressing PMS/PMDD should be prioritized as a public health issue to support the health and empowerment of Afghan women.

## Data Availability

The data that support the findings of this study are available from the corresponding author, M.R., upon reasonable request. The data are stored in SPSS format and can be provided electronically. For assistance in accessing the data, please contact mramozi2019@gmail.com

## Abbreviations Used

PMDD: Premenstrual Dysphoric Disorder
PMS: Premenstrual Syndrome
PTSD: Post-Traumatic Stress Disorder
